# Low Neutrophil Counts in Milk Are Associated with an Increased Frequency of Antimicrobial Treatments

**DOI:** 10.3390/pathogens14111104

**Published:** 2025-10-29

**Authors:** Alfonso Zecconi, Valerio Sora, Emanuele Invernizzi, Francesca Zaghen, Viviana Chierici Guido

**Affiliations:** 1 Department of Biomedical, Surgical and Dental Sciences, School of Medicine, University of Milano, Via Pascal 36, 20133 Milan, Italy; valerio.sora@unimi.it (V.S.); francesca.zaghen@unimi.it (F.Z.); 2 Distretto Veterinario Basso Lodigiano, ATS Città Metropolitana di Milano, Distretto Veterinario Basso Lodigiano, Via Trieste 76, 26845 Codogno, Italy; einvernizzi@ats-milano.it; 3 Department of Clinical and Community Sciences, School of Medicine, University of Milan, Via Celoria 22, 20133 Milan, Italy; 4 Associazione Regionale Allevatori Lombardia, Via Kennedy 30, 26013 Crema, Italy; v.guido@aral.lom.it

**Keywords:** milk, leukocytes, immunity, neutropenia, somatic cells, antimicrobial treatment, disease risk

## Abstract

Several studies have demonstrated an association between impaired innate immunity and metabolic parameters, particularly during the periparturient period. However, to our knowledge, no study has been conducted under field conditions investigating the link between low milk polymorphonuclear neutrophil (PMN) levels and increased disease frequency. In an attempt to address this knowledge gap, this study examined 6209 cows from 20 dairy herds in Lombardy that were enrolled in a monthly individual dairy herd improvement milk testing program. Analyses of milk test record samples (MTR) included somatic cell count (SCC) and differential cell count (DSCC). A third variable, PLCC (polymorphonuclear neutrophil leukocyte cell count), was calculated by multiplying SCC × DSCC, thus representing PMN cells/mL. A database including compulsory records of all antimicrobial treatments applied in each herd was used as a proxy for disease frequency. In total, 58,090 valid MTR and 12,014 antimicrobial treatments (AMT) were considered for this study. Statistical analyses showed a significant association between the prevalence of cows with a low number of milk PMN and the prevalence of AMT. These results allow routine identification of whether the number of cows with low PLCC exceeds an alarm level within a herd. This threshold was calculated using an ROC curve with a cut-off point of 6% for AMT. This threshold was estimated at 2%, providing 78% accuracy in identifying herds at risk of an increasing treatment rate. This study confirms that cellular markers measured within MTR systems are useful in identifying herds at risk of impaired cellular immunity, thus paving the way for further studies assessing herd and cow immune status with routine milk sampling.

## 1. Introduction

The increasing and serious concerns regarding the spread of antimicrobial resistance (AMR) in both human and veterinary medicine have renewed interest in developing new approaches to prevent and control infectious diseases, including enhancing innate immune responses [1,2,3,4]. These aspects are not new in the context of the bovine mammary gland. Indeed, interest in the immune response of the mammary gland and ways to improve it has been evident since the 1990s [5,6,7,8]. Unfortunately, despite the large number of studies on this topic, there have been no practical applications [8]. This may be related to the difficulty of measuring cow immune status, and especially cellular immunity, in a practical and sustainable way, impairing the assessment of the efficacy of the different approaches. Indeed, while a number of tests for innate immunity at the blood level are available [9], these tests, in the food-producing animals context, are generally expensive and time-consuming. Therefore, only studies involving a limited number of animals are feasible, and it could be questionable to generalize their results to the cow population. These problems become even more severe when these markers are measured in milk. Most tests used for blood analysis are not suitable for milk due to its composition, particularly the presence of fat. Although some markers of the innate immune response are available, they are either expensive or inapplicable under field conditions as well [6,10]. Among the very few markers available, somatic cell count (SCC) is sustainable and is very accurate in diagnosing subclinical mastitis but performs poorly as a marker of cellular immunity [11,12]. Much effort has been devoted to investigating differential cell counts in milk because the leukocyte formula, as demonstrated in blood, is a highly useful and consistent marker of cellular immunity [9]. However, until recently, investigations on milk differential cell counts (DSCCs) were limited by poor reproducibility and the high cost of available methods. This limited their application to studies with relatively small sample sizes [11,13,14,15].

The development of a high-throughput tool for performing DSCC has paved the way for the sustainable exploration of cow innate immunity under field conditions. Most of these studies aimed to apply DSCC in the diagnosis of subclinical mastitis and/or an increased risk of mastitis, and the results confirmed the usefulness of this approach [16,17,18,19,20,21,22]. The “classical” approach is based on the definition of a threshold and involves applying either SCC or DSCC as markers. Values of SCC and DSCC over the specific threshold indicate the presence of subclinical mastitis [23,24].

The presence of polymorphonuclear neutrophils (PMN) in the milk of healthy glands seems to correlate inversely with the risk of intramammary infections or a reduction in the severity of clinical mastitis [5,25,26]. However, the same measurements may indicate the opposite condition: the presence of an insufficient amount of PMN [27,28]. Indeed, in the healthy gland, PMN have the capability to migrate from peripheral blood through endothelial gaps in the mammary epithelium to milk. Thus, the normal uninfected mammary gland is supplied with a constant source of PMN, stemming from cells maturing in the bone marrow, then released into the circulation, where they spend approximately 9 h before migrating into tissue [5,8]. In healthy cows, the production and destruction of PMN is tightly regulated, which keeps their number in blood, milk, and tissue constant. Since the number of PMN in milk, in the absence of an inflammatory process, is regulated by their concentration in the bloodstream and their release from the bone marrow, very low levels may be a sign of transient or chronic immune impairment [9].

Several experimental studies have shown an association between innate immunity impairment and metabolic parameters, particularly during the periparturient period [29,30,31]. However, to the best of our knowledge, no study has been conducted under field conditions on the association between low levels of PMN and a higher frequency of antimicrobial treatments (AMT) related to the presence of an infectious disease. This information is lacking due to the inability to measure PMN in milk, as described before, as well as the difficulty of objectively assessing disease frequency based on herd records.

In an attempt to address this knowledge gap, this study aims to demonstrate the existence of an association between the prevalence of milk PMN values below a defined threshold, as measured by monthly milk test record samples (MTR), and the frequency of AMT based on compulsory treatment recording practices in Italy.

## 2. Materials and Methods

### 2.1. Herd and Cow Selection

This study considered 6209 cows from 20 dairy herds in Lombardy that are enrolled in the Italian Breeder Association (AIA) monthly individual DHI MTR. The herd size was in the range of 84–900 lactating cows, and 95% of the cows were Italian Friesian.

Overall, 58,090 valid MTR and more than 83,000 recorded treatments were available. Among these latter ones, 12,014 concerned AMT, and they were considered for this study. Within the AMT, 4212 were related to mastitis treatment during lactation. Antimicrobial treatments at drying-off were not considered in the study.

### 2.2. Sample Collection

Individual cow samplings were performed by certified methods by means of Lactocorder™ (WMB AG, Balgach, CH, Switzerland), delivered refrigerated to the Regional Breeders Association of Lombardy (ARAL) labs on the same day, and analyzed within 30 h of sampling.

### 2.3. Cellular Marker Analyses

Milk analyses on MTR samples included SCC and DSCC and were carried out on Fossomatic™ 7DC (Foss A/S, Hillerød, DK, Denmark). The DSCC was assessed by the method described by Damm et al. [16]. This method allows us to identify within a milk sample the macrophages and the combination of PMN and lymphocytes (LYM). DSCC is expressed as the combined proportion (%) of PMN and LYM in the overall count of milk cells. Cow and MTR data collected in 2024 for all 20 herds considered were recorded in a database.

### 2.4. Treatment Records

All treatments applied to dairy cows in Italy must be compulsorily recorded (Regulation EU 2019/6 of the European Parliament and of the Council) in an electronic database included in Classyfarm, an integrated surveillance system of Italian livestock farms [32]. From this database, all antimicrobial treatments applied in 2024 in the 20 herds were retrieved and the data were combined with the database related to MTR data.

### 2.5. Definition and Description of Herd Immune Status

The variable PLCC (polymorphonuclear neutrophil leukocyte cell count) was used to define the immune status of the herd. It was calculated by multiplying SCC × DSCC. This variable represents the total number of PMN + LYM/mL [17,18]. This variable represents the amount of PMN in milk, since the variability of this marker is mainly due to changes in the proportion of PMN, while the proportion of LYM is relatively constant [16,20].

If we define a healthy cow as having an SCC in the range of 10,000–50,000 cells/mL and a DSCC of 60% [20], the PMN concentration will be approximately 6000–30,000 cells/mL. In both human and veterinary medicine, neutropenia is defined as a PMN level below baseline values [33,34]. Based on these considerations, we assume that a PLCC value below 5000 cells/mL is a conservative threshold for defining cows at risk of having an inadequate level of PMN (neutropenia), and this level was considered for further analysis.

### 2.6. Statistical Analysis

The final database includes herdID, cowID, number of antimicrobial treatments, SCC, DSCC, and PLCC. SCC and PLCC have a non-normal distribution; therefore, their values were log-transformed.

The statistical analyses included correlation, linear regression analysis, and ROC curve; they were all performed using the related procedures of SPSS 29.0.1 (IBM Corp, Armonk, NY, USA).

For what concerns the ROC curve, the following parameters were calculated for each specific threshold of AMT frequency:

- Accuracy: expressed as the proportion of correctly classified subjects [true positive (TP) + true negative (TN)] among all subjects.
- Sensitivity (Se): the proportion of TP/[TP + false positive (FP)]
- Specificity (Sp): the proportion of TN/[false negative (FN) + TP]
- Positive predictive value (PPV): TP/(TP + FN)
- Negative predictive value (NPV): TN/(TN + FP)
- Positive likelihood ratio (LR+): [TP/(TP + FN)/FP/(FP + TN)]
- Negative likelihood ratio (LR−): [FN/(TP + FP)/[TN/(TN + FP)]

## 3. Results

### 3.1. Data Description

**Figure 1:**
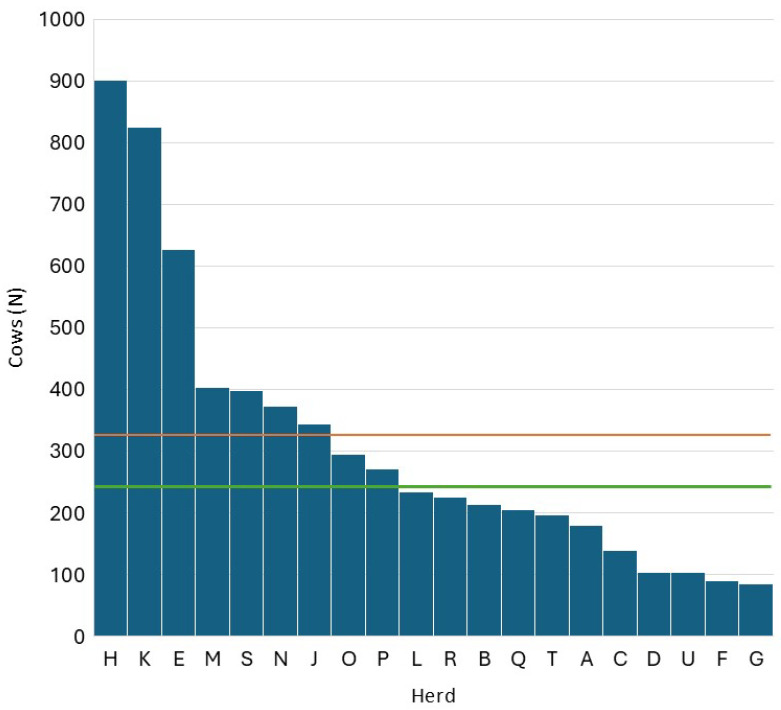
Distribution of herd size within the 20 herds considered in Lombardy (the red line represents the mean, and the green line represents the median).

Figure 1 reports the distribution of herd sizes in the 20 herds considered. The mean herd size was 310 cows/herd, which is higher than the Lombardy average at the end of 2024 (108 lactating cows/herd). The difference between the two means is due to the characteristics of the herds enrolled in ARAL, which include most of the largest and most efficient herds in the region.

**Figure 2:**
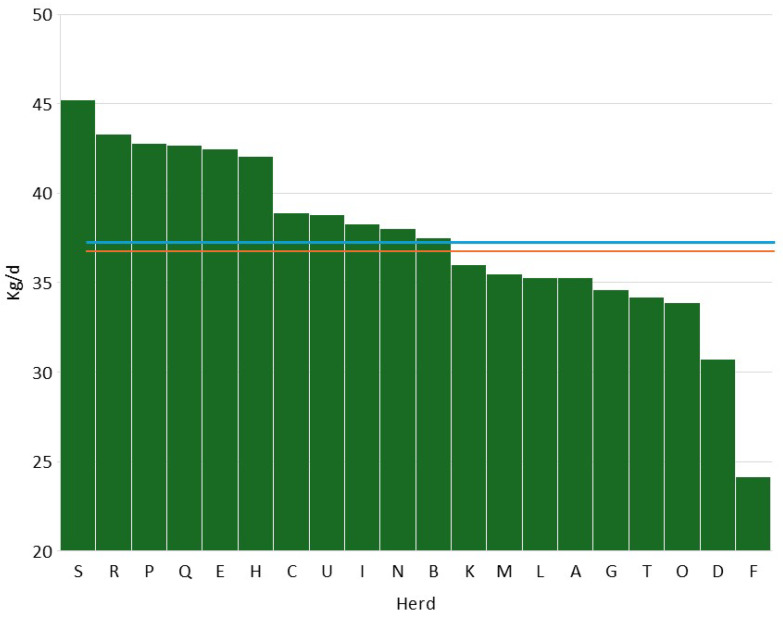
Distribution of average daily production in the 20 herds considered in Lombardy (the red line represents the mean, and the green line represents the median).

Figure 2 describes the distribution of average daily milk yield during 2024. The mean was 37.5 kg/d, a value very close to the median (37.8 kg/d). Also, in this case, the values are higher than the Lombardy average (28.9 kg/d).

Table 1 summarizes the distribution of SCC, DSCC, and PLCC variables in the 20 herds considered, showing a maximum mean value for SCC of 5.13 Log_10_ cells/mL (≈135,000 cells/mL) and a minimum of 4.62 Log_10_ cells/mL (≈33,000 cells/mL). DSCC varied between 55.94% and 66.44%, while PLCC ranged between 4.22 Log_10_ cells/mL (≈16,500 cells/mL) and 4.98 Log_10_ cells/mL (≈95,500 cells/mL). The values of these markers suggest that the 20 herds considered have good herd management and a good level of udder health.

### 3.2. Antimicrobial Treatments

**Figure 3:**
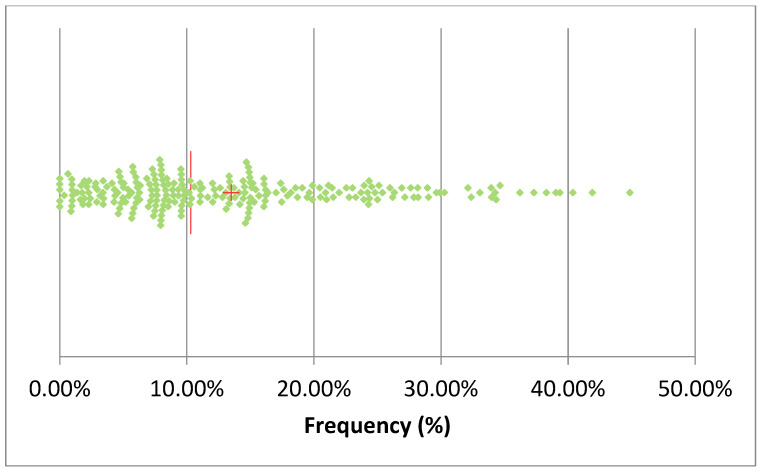
Scatterplot of the monthly frequency of treatments/cows in the herd in the 20 herds considered (the red line represents the median, and the cross represents the mean). A single sample with a frequency of 70% was omitted.

**Figure 4:**
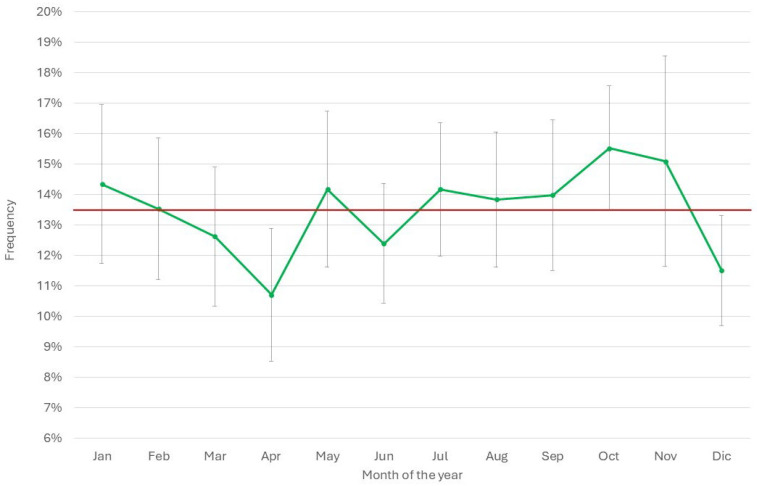
Distribution of the monthly mean (±std. err.) frequency of treatments in the 20 herds considered by month of the year 2024. The red line represents the overall mean of the year.

Table 2 summarizes the total number of AMT in 2024 and the proportion of mastitis treatments among them. The total AMT varied between 17 (herd U) and 2695 (herd K), while mastitis antimicrobial treatment (MAT) frequency during lactation varied between 4% (herd K) and 96% (herd R) of the total AMT. The median of mastitis antimicrobial treatments was 55%, while in 25% of the herds, the MAT frequency was below 35% of the total AMT. The monthly median of the frequency of AMT per cow in the herd (AMT/CH) was 10.3% (Figure 3), and in 50% of the herds, AMT/CH frequency was in the range of 12–70%. The mean was, obviously, higher (13.5%), and the first quartile was 6%, while the third was 18.8%, suggesting that the overall frequency of AMT/CH was relatively low on a monthly basis. This observation was confirmed by the distribution of treatments by month of the year, ranging between 10% and 16% (Figure 4), with lower values in April and December, while higher values were observed in October and November.

**Figure 5:**
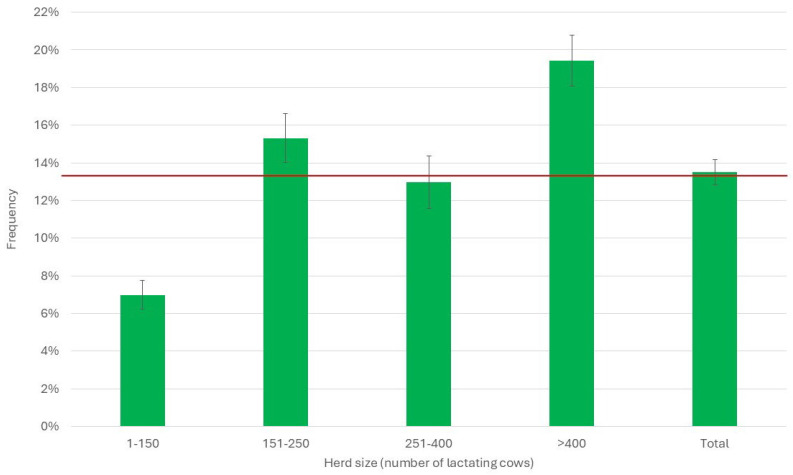
Distribution of the monthly mean (±std. err.) frequency of treatments/cows per herd in the 20 herds considered by herd size. The red line represents the overall mean of the year.

The distribution of AMT/CH by herd size (Figure 5) showed that the smaller herds had the lowest mean frequency (7.0% ± 0.8%), while in larger herds, the values were always over 10%, with the highest in the largest herds (19.45 ± 1.3%).

### 3.3. Herd Immune Status

**Figure 6:**
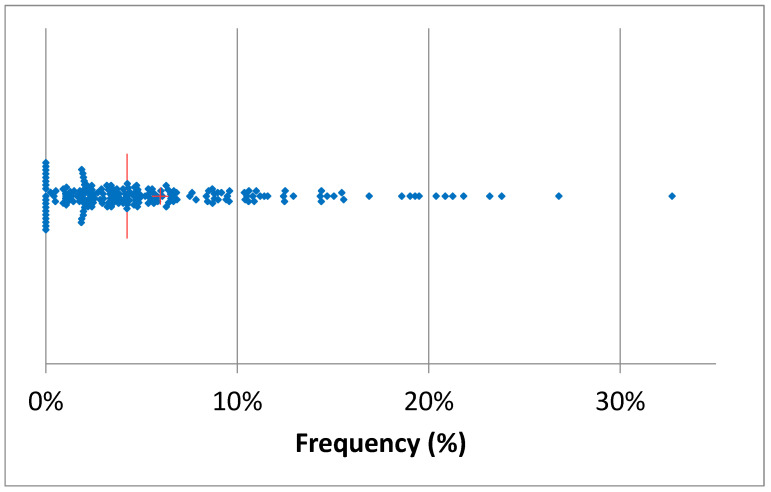
Scatterplot of the monthly mean frequency (±std. err.) of cows with PLCC < 5000 cells/mL in the 20 herds considered (the red line represents the median, and the cross represents the mean).

The distribution of the frequency of cows with PLCC < 5000 cells/mL is shown in Figure 6. The median of the distribution is 4.2%; therefore, 50% of the herds had higher frequency of cows below the threshold, and 25% of them had a frequency ≥8.6%.

**Figure 7:**
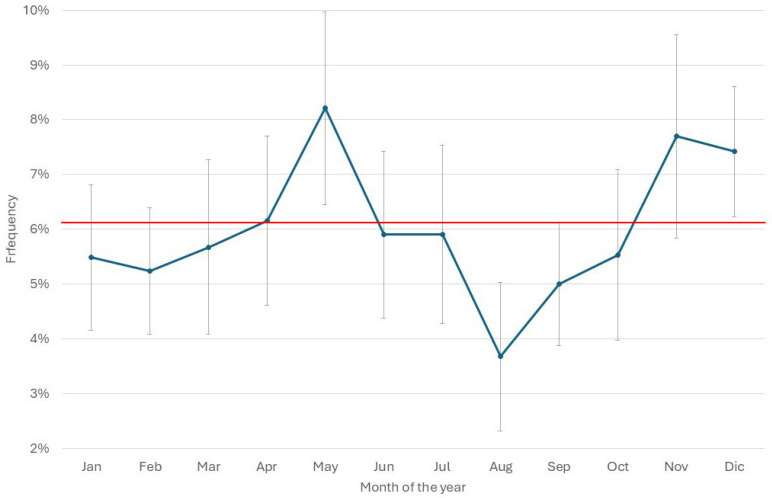
Distribution of the monthly mean (±std. err.) frequency of cows with PLCC < 5000 cells/mL in the 20 herds considered by month of the year 2024. The red line represents the overall mean of the year.

During 2024, the frequency of cows below the threshold varied between 3.7% (August) and 8.2% (May), with higher frequencies during the winter months (November and December) (Figure 7).

**Figure 8:**
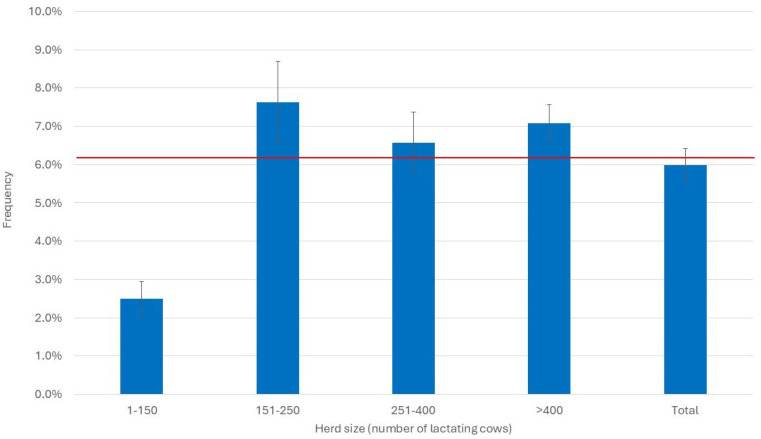
Distribution of the monthly mean (±std. err.) frequency of PLCC < 5000 cells/mL in the 20 herds considered by herd size. The red line represents the overall mean of the year.

Herd size also affected the frequency of cows below the PLCC threshold. Indeed, the smaller herds had a mean below 2.5%, while in the larger herds, the means were close and within the range of 6.6–7.6% (Figure 8).

### 3.4. Correlation Between Immune Status and Frequency of Treatments

The objective of this study was to investigate the presence of a correlation between the frequency of cows below the PLCC threshold and the frequency of treatments as an indicator of reduced immune competence, leading to an increase in the frequency of disease.

The initial step in the research process entailed the examination of whether the cellular pattern exhibited by the cows fell below or above the established threshold. The findings indicated that low PLCC values were associated with a low proportion of PMN (see Table 3). Notably, the proportion of PMN in samples with PLCC values < 5000 cells/mL was nearly half of that observed in the high PLCC samples. It is obvious that SCC levels are diminished in cows with a PLCC count < 5000 cells/mL, and these values are widely regarded as an indicator of optimal udder health. The frequency of samples falling below the established threshold is low, though not negligible, accounting for 6.5% of the total. These findings support the hypothesis that this condition does not have a physiological basis.

To assess the presence of a correlation between the frequency of AMT/CH and PLCC < 5000 cells/mL, the Pearson’s correlation coefficient was calculated. The results showed that the coefficient was 0.44 (95% CL 0.31–0.55), which is statistically significant (*p* < 0.0001), confirming the presence of a significant correlation.

**Figure 9:**
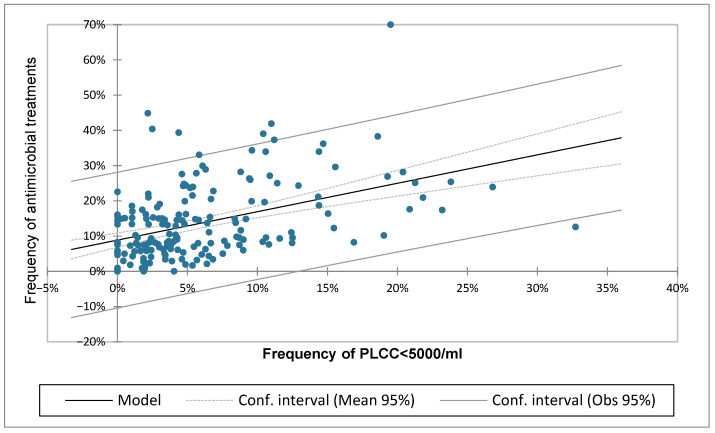
Linear regression model calculated for the frequency of cows with a polymorphonuclear neutrophil leukocyte cell count (PLCC) of < 5000/mL and the frequency of antimicrobial treatments per cow in the herd.

The linear regression analysis is reported in Table 4 and Figure 9. The statistical analysis confirmed the presence of a significant association between the two parameters, with a regression coefficient of 0.19 and a linear increase of AMT/CH as the frequency of cows with PLCC < 5000 increased.

This result also suggests that identifying a threshold for the frequency of PLCC < 5000 cells/mL may serve as a method for defining a herd as being at risk of an increased number of treatments. To define this threshold, the ROC was calculated, defining as the response variable two different levels of frequency of AMT/CH—6% and 10%—representing, respectively, the lower 25th percentile and the median of the monthly AMT/CH frequency in the 20 herds considered.

The results are reported in Table 5, and they show that the ROC curve related to the 10% AMT/CH threshold gave relatively poor results, with an accuracy of only 67% and values for sensibility and specificity of 65% and 69%, respectively. The 6% AMT/CH threshold gave more interesting results, having an accuracy of 78% and higher values for sensitivity (85%), while specificity was lower (57%). Practically, when in a herd the frequency of samples with PLCC below 5000 cells/mL is over 2%, we may expect, in 85% of the cases, a level of AMT > 6%. On the other hand, a herd with a frequency of PLCC samples <5000 cells/mL is >2% and has an 86% probability of treating more than 6% of the cows (positive predictive value). If the PLCC frequency is >2%, there is a 54% chance that the herd also has <6% of treatments (negative predictive value).

## 4. Discussion

Modern, sustainable dairy farming must address a variety of issues raised by civil society, health authorities, and processors. One of the most important of these is reducing the use of antimicrobials to slow the spread of antimicrobial resistance [35,36]. This is particularly important when considering dairy production from a One Health perspective. Reducing antimicrobial usage can be achieved through various approaches, such as improving and accelerating diagnosis, implementing better management, and raising hygiene standards. However, the approach with the greatest potential is increasing the animal’s immune status [37,38]. All of these new approaches require tools that allow rapid and efficient changes to overall farm management, as farms are constantly growing in size. One of these tools has been in use for a long time. It consists of the DHI approach through the MTR system and is carried out in Italy by the National Breeders’ Association (AIA). While this tool is not new, it has evolved over time thanks to new technologies that have made it possible to supplement the classic parameters (cells, fat, proteins, and lactose) with many others that provide information on metabolic and health aspects. A fundamental step forward in this regard is the ability to perform a differential count of milk cells. Several papers [17,22,23,24,39] have shown that the assessment of DSCC is accurate and efficient in defining udder health. It is also effective in assessing the effects of different management systems and changes in milk composition [19,40].

### 4.1. How to Measure Herd Immune Status

While the capability of DSCC to assess the inflammatory status of the gland is well understood, the ‘other side of the coin’, namely the assessment of the cow’s immune status, remains unexplored. As previously mentioned, the production and destruction of PMN are tightly regulated in healthy cows, keeping their numbers in the blood, milk, and tissues constant. Therefore, we can assume that PMN concentrations in milk, as measured by PLCC, are a proxy for the amount of circulating PMN and that lower levels in milk indicate neutropenia or impaired migration from the bloodstream. Such impairment could be considered a risk factor for an increased occurrence of infectious diseases. The status of neutropenia or impaired cellular immunity was defined using an approach that has been successfully utilized in human medicine. This approach defines neutropenia as PMN values falling below the lower limit of the physiological range for a given species. Healthy cows were shown to have a PMN content in the range of 6000–30.000 cells/mL [20]. Consequently, a value of PLCC < 5000 cells/mL was designated as the optimal threshold for an inadequate level of PMN. This paper aims to confirm this hypothesis, supporting the use of PLCC as a marker of immunity to identify herds or cows at risk.

### 4.2. Antimicrobial Treatments

One way to verify this hypothesis would be to observe an increase in AMT resulting from an increase in the incidence of infectious diseases, alongside a decrease in the number of PMN. However, the accuracy of disease recording is often uncertain, particularly but not exclusively, in Italian dairy herds. The recent implementation of Regulation (EU) 2019/6 of the European Parliament and of the Council has made it mandatory to record antimicrobial treatments administered on Italian livestock farms in an official electronic database. This approach provides reliable data, independent of the breeder’s willingness, ability, and availability to record pathologies, which has always been a problem with this type of survey. Therefore, we have obtained consistent and accurate information on diseases requiring antimicrobial treatment, together with accurate monthly measurements of milk cells (total and differential).

### 4.3. Correlation Between Herd Immune Status and Frequency of Antimicrobial Treatments

This study examined 20 dairy herds in Lombardy that were characterized by larger herd sizes and lower somatic cell counts (SCC) than the regional average. These herds were selected to represent the best examples of optimal management, performance, and health. Consequently, they stand to benefit the most from consistent, precise monitoring of their immune status. In these herds, AMT was applied to between 12% and 70% of lactating cows each year. The lowest observed frequencies were exhibited by smaller herds, which was an unexpected finding. One potential explanation for this phenomenon is the increased prevalence of voluntary culling, occurring at a rate of 36% compared to 26% in larger herds [41]. This practice is driven by the desire to obtain the financial incentives associated with the European Common Agricultural Policy, which are restricted to herds that reduce antimicrobial treatments.

With regard to the PLCC values, the results showed that 6.5% of the samples were below this value, with peaks of around 8% occurring in May, November, and December. Furthermore, smaller herds exhibited the lowest frequencies (<3%), likely for the same reasons that have been posited for AMT frequencies (an increase in the culling rate). Conversely, the lowest frequency of low PLCC cows was documented in August, coinciding with the period of peak heat stress in Lombardy. This suggests that the impact of heat stress on PMN levels is less severe than previously hypothesized [42,43]. The findings suggest that the reduction in PMN levels is mainly attributable to other factors, including infection, changes in management practices, and metabolic diseases, among others [44].

The statistical analyses performed demonstrated a significant association between the prevalence of cows with a low proportion of PMN in milk and an increased prevalence of AMT/CH, not necessarily related to the treatment of mastitis. In fact, both Pearson’s correlation coefficient and linear regression analysis demonstrated a positive correlation. The existence of a statistically significant and positive association between the frequency of cows with a PMN content < 5000 cells/mL and the number of AMT administered suggests that a low PMN content in milk may be indicative of a cow’s diminished immune competence. In practice, the increase in the frequency of PLCC < 5000 cells/mL is assumed as a status of neutropenia or, more generally, of an impairment of nonspecific immunity, leading to an increase in diseases and, consequently, in AMT.

These results suggest that it is possible to routinely identify if the number of cows with low PLCC surpasses an alarm level within a herd. The calculation of this level was performed using the ROC curve, with a threshold of 6% for AMT/CH (the level of the 25th percentile of herds with lower AMT frequency). The calculated level was established at 2%, which, with an accuracy of 78%, enabled the identification of a herd exhibiting a heightened risk of requiring treatment. The level was determined in a specific group of herds, not randomly selected, which may not be representative of the population in other regions or with different management practices. However, the results of this study demonstrate the efficacy of measuring the cellular markers in MTR, combining them in the PLCC variable, and using the latter to identify herds at risk of impaired cellular immune response.

Further studies are necessary to substantiate these results and to verify whether the assessment of immune status at the herd level can also be applied to individual cows. The objective of such an assessment would be to identify cows that are at risk of developing diseases due to an impaired immune response.

## 5. Conclusions

The capacity to discern an ineffective immune response at the herd level will empower farmers and their advisors to implement the measures they deem most suitable to restore the animals’ immune response to an optimal level. The results of the present study indicate the existence of a sustainable method that has the potential to signal a risk condition at the herd level. Further studies are required to corroborate these findings on a broader population. Nevertheless, the results of this study indicate that the presence of this risk condition may necessitate the implementation of specific measures. These measures are expected to enhance animal welfare, reduce the utilization of antibiotic treatments, and, more broadly, increase the overall sustainability of the farm.

## Figures and Tables

**Table 1:** Descriptive statistics (mean ± standard deviation) for the three cellular markers considered (somatic cell count—SCC, differential cell count—DSCC, and polymorphonuclear neutrophil leukocyte cell count—PLCC).

Herd	SCC (Log_10_/mL)		DSCC (%)		PLCC (Log_10_/mL)	
	Mean	Std. Dev.	Mean	Std. Dev.	Mean	Std. Dev.
A	5.07	0.61	61.50	17.42	4.83	0.71
B	4.87	0.63	60.55	17.53	4.63	0.73
C	4.68	0.56	57.30	18.55	4.43	0.68
D	4.99	0.62	59.92	17.54	4.74	0.72
E	4.85	0.63	61.61	17.97	4.62	0.73
F	5.13	0.63	64.59	17.32	4.92	0.74
G	5.06	0.59	66.42	15.43	4.85	0.68
H	4.95	0.63	63.44	18.08	4.73	0.74
K	4.72	0.58	61.07	17.54	4.48	0.68
J	5.02	0.67	65.18	18.64	4.81	0.77
L	5.04	0.67	66.44	17.45	4.85	0.76
M	5.04	0.69	66.09	17.12	4.84	0.80
N	4.84	0.62	63.15	17.53	4.62	0.72
O	5.00	0.62	64.93	16.23	4.80	0.72
P	4.74	0.63	58.55	19.44	4.48	0.75
Q	4.52	0.55	52.89	18.88	4.22	0.66
R	4.70	0.57	55.94	18.42	4.43	0.67
S	4.74	0.55	56.15	18.08	4.46	0.65
T	5.13	0.63	72.17	14.77	4.98	0.70
U	4.97	0.60	63.73	16.25	4.76	0.69
Total	4.88	0.64	61.84	18.10	4.64	0.74

**Table 2:** Total number of antimicrobial treatments in the 20 herds considered in Lombardy, and the proportion of mastitis antimicrobial treatments.

Herd	Total Antimicrobial Treatments (N)	Mastitis Treatments Proportion
A	531	27%
B	308	56%
C	242	58%
D	86	79%
E	1865	13%
F	41	37%
G	81	68%
H	1829	40%
K	2695	4%
J	339	74%
L	152	84%
M	618	73%
N	417	65%
O	111	70%
P	1015	54%
Q	642	23%
R	346	96%
S	444	46%
T	235	46%
U	17	29%
Total	12,014	35%

**Table 3:** Somatic cell count (SCC) and differential cell count (DSCC) mean values (±Std. Dev.) in cows classified by PLCC (polymorphonuclear neutrophil leukocyte cell count) levels.

PLCC Status	SCC		DSCC		Proportion of Samples
	Mean	Std. Dev.	Mean	Std. Dev.	
Below 5000 cells/mL	4.0	0.16	35.4	11.3	6.5%
Over 5000 cells/mL	4.9	0.61	63.8	16.9	93.5%

**Table 4:** Results of linear regression analysis on the frequency of cows with a polymorphonuclear neutrophil leukocyte cell count (PLCC) of <5000 cells/mL.

Model	Coefficient		*p*	95.0% Confidence Interval	
	B	Std. Err		Lower	Higher
Constant	0.089	0.010	<0.001	0.068	0.109
PLCC < 5000/mL freq.	0.807	0.122	<0.001	0.566	1.048

**Table 5:** Results of the ROC curve analysis applying an antimicrobial treatment (AMT/CH) threshold of 6% and 10% for the response variable, and the frequency of samples with polymorphonuclear neutrophil leukocyte cell count (PLCC), cells/mL, as a test variable.

Calculated PLCC Threshold	2.0%	4.4%
Parameter	AMT/CH > 6%	AMT/CH > 10%
Sensitivity	85.0%	64.9%
Lower bound (95%)	78.0%	54.8%
Upper bound (95%)	90.0%	73.8%
Specificity	56.8%	68.9%
Lower bound (95%)	42.2%	58.7%
Upper bound (95%)	70.3%	77.5%
Positive predictive value	86.2%	68.5%
Negative predictive value	54.3%	65.3%
Positive likelihood ratio	1.97	2.09
Negative likelihood ratio	0.26	0.51
Accuracy	78.3%	66.8%

## Data Availability

The data are not publicly available due to privacy regulations concerning MTR.

## Abbreviations

The following abbreviations are used in this manuscript:

AMT	Antimicrobial treatment
AMT/CH	Antimicrobial treatment/cows in the herd
DHI	Dairy herd improvement
DSCC	Differential somatic cell count
MTR	Milk test record
PLCC	Polymorphonuclear neutrophil leukocyte cell count
PMN	Polymorphonuclear neutrophils
SCC	Somatic cell count
